# Against the right to mutilation: an analogy ad absurdum

**DOI:** 10.1007/s40592-025-00279-6

**Published:** 2026-01-07

**Authors:** Morten Dige

**Affiliations:** Department of Philosophy and History of Ideas, Aarhus University, Jens Chr. Skous Vej 7, Aarhus C., 8000 Denmark

**Keywords:** Amputation, Bodily harm, Euthanasia, Healing professions, Killing, Moral prohibitions

## Abstract

A particular repertoire of arguments against the legalization of euthanasia has been part of the philosophical debate for more than sixty years. It has been repeated and criticized many times over the years, but I try in this article to show that the arguments are flawed by way of a different argumentative strategy that I propose to call *analogy ad absurdum*. I thus argue that patients as well as the medical profession would be best served with a total prohibition on amputations. My arguments closely follow analogous well-established and well-reputed arguments in the debate over euthanasia. To the extent that those arguments establish that we ought not to accept killing as an integral part of the healing profession, we ought to be equally dismissive of the medical practice of amputations. Conversely, if we find that the worries about and objections to the practice of amputations could most probably be handled, we should be skeptical about the analogous arguments concerning euthanasia.

## Introduction

I aim in this article to challenge several stock objections to legalized euthanasia. Most, if not all, of them go all the way back to a 1958 article by Yale Kamisar and have been repeated many times since (Kamisar 1958). I challenge them by way of a strategy for which I propose the term “analogy ad absurdum”. The strategy is to show how arguments analogous to the arguments against euthanasia seem absurd or at least far-fetched. I want to stress that such analogies ad absurdum present the objections against euthanasia with a *challenge*, not a knock-down argument. I will finish the article by considering possible ways to invalidate or counter the analogy.

I analogize euthanasia to amputations. It should be obvious how amputations are analogous to euthanasia in ways relevant for an ethical evaluation of those practices. They are both considered to be at the margins (at best) of good medical practice, something to be resorted to as a last resort only. They both involve the use of medical methods and technologies in ways that harm the person exposed to them. By implication, they involve violations of strong, well-established moral prohibitions, namely “do no harm” and “do not kill”, respectively.

The “do no harm” (*primum non nocere*) principle is often highlighted as essential to or constitutive of medical ethics. Even so, amputations, i.e., the severing of limbs from patients, is a routine practice in hospitals around the globe. The numbers are staggering. Experts estimate that on a global scale a leg is amputated every 30 s. The numbers are even rising, not least due to the increase in diabetes patients who are far more susceptible to infections in lower extremities. The rate of amputations in the US increased by 50% between 2009 and 2015 up to 4.6 for every 1000 adults (Geiss et al. 2019). Not included in these figures are the procedures cancelled in the last minute, so a further unknown number of patients have been put at risk of being amputated and have had to consider whether they would be allowed to retain their limbs or not.

In the following, I will argue (ad absurdum) that we would be better served with a total prohibition on amputations. Some will foreseeably object that there are patients whose lives can be saved only by amputations. Would it not therefore be morally required to grant such patients a right to amputation (or to be frank: a right to mutilation) as a last resort? My answer is that even though there might be isolated cases in which amputation seems to be the only acceptable solution for the patient, the direct and indirect moral costs of admitting amputation as an integral part of health care far outweighs the alleged benefits. My arguments closely follow analogous well-established and well-reputed arguments in the debate over euthanasia (as will appear from the references). To the extent that those arguments establish that we ought not to accept killing as an integral part of the healing profession, we ought to be equally reluctant to accept maiming as an integral part of the healing profession. I have identified eight such reasons which will be laid out in the following.

## “Amputation” is a misleading euphemism

Let us first be clear that we are talking about a serious and irrevocable procedure. Once a limb has been severed from the body, we cannot very well attach it again. Now, the very word “amputation” has a certain clinical and clean ring to it (*putare* means to clean). What it really covers, however, is an invasion in bodily integrity, a mutilation of the body. This fact cannot be altered by an informed consent from the person being mutilated. Consequently, most people, when asked whether they find amputations ethically permissible, do not fully realise what they give an answer to. One may wonder whether they would be as tolerant when they come to realise that it involves a doctor dismembering a patient’s body (see Hartling 2021a: 20–25 for analogous arguments about euthanasia). To witness all the gory, bloody details of the procedure and the formidable weaponry involved would make most people think twice about continuing this grotesque practice.

## Disregarding the ban on bodily harm

Apart from the ban on killing, the ban on physical assault is arguably the most basic moral prohibition we have. The ban serves an extremely important symbolic function in the service of a society of justice and humanity. This is the reason why the prohibition cannot be nullified by consent. Our society does not condone maiming as a relationship between two legally competent, consenting persons just as it does not accept killing as a lawful relation between consenting persons (Hartling 2021b). I consider the disturbing slippery slope perspectives of relativizing the ban on bodily harm below. The claim at this point is that harm is morally wrong in and of itself. The ban further protects a basic right to bodily integrity, a right protecting a value that cannot be rendered nugatory by the entirely predictable evils of bodily infirmity that sooner or later afflicts us all (Gormally 1994: 772).

## Neither for love nor money: Doctors must not maim

If we acknowledge that harm is morally wrong in and of itself, surely it ought not to be made part of routine medical practice. This issue touches the medical profession at its very core. Just as doctors should not kill, neither for love nor money (Kass 1989), we should keep in mind that it is central to the medical vocation to be a *healer*. Healing is about pursuing the wholeness of the body, not to prune it. Medicine should side with bodily integrity, not disintegrity. A physician true to her calling would never depart from the taboo against harming her patient. For all its fascinating powers, modern medicine should show restraint in order not to put those powers in the service of maiming and killing. Maybe Hippocrates was on to something important when he denied surgeons access to the holy order of physicians? Patients should go elsewhere than the medical profession if they think the answer to their suffering is to be maimed by another. To paraphrase Daniel Callahan: too often in history, violence has seemed the quick, efficient way to put aside that which burdens us (Callahan 1992). If we allow patients the right to have their bodies maimed by doctors it surely is self-determination run amok.

## Legal discrimination between worthy and unworthy limbs

As correctly pointed out by the Danish Ethical Council, a state’s legislative power ought not to be involved in “demarcating lives not worthy of preservation” (Danish Ethical Council 1996: 129). However, having amputations as a lawful practice means that the legislative power deems some limbs more worthy of protection and preservation than others. In medical practice, the upshot is that doctors would have to assess whether e.g., the patient’s leg is worth preserving. The justification for amputations thus seems to be borne on the premise that certain patients’ mobility is not worth preserving just as the justification for “assisted dying” (i.e., physicians killing patients) is ultimately borne on the premise that certain lives are not worth living (Hartling 2021b: 2). But neither lawmakers, nor doctors should decide who are entitled to intact bodies and who not.

## Choosing the easy way out

Having the very option of amputation as a routine part of medicine provides an easy – all too easy – solution to medical problems. If an arm or a leg gets infected or does not function optimally any longer, we have amputations at hand as a quick fix. But it counts as a medical failure, if anything does, to “heal” a sick leg or a foot by discarding it altogether. A possible, in fact predictable, effect of having the quick, easy (and cheap) solution is that other, much gentler, *healing* solutions are neglected or under-prioritized. And the sad fact is that such alternatives do exist. As demonstrated by Bakker et al., foot infections can in large measure be prevented by:

- Regular inspection and examination of the at-risk foot.
- Identification of the at-risk foot.
- Education of patient, family, and healthcare providers.
- Appropriate footwear.
- Treatment of nonulcerative pathology (Bakker et al. 2012).

All this, however, calls for complex, time-consuming, and expensive work procedures. Since patients suffering from diabetes and sequela are generally not among the most socioeconomically advantaged and vocal, one might worry that they are just fobbed off with amputations instead. A report from 2021 shows significant differences in amputation levels across regions in Denmark and one cannot help wondering whether the easy, legal access to amputations have made health care providers in some regions more careless about their patients’ right to bodily integrity (Albinus 2022). A total ban on amputations would foster stronger attention to preventive measures.

A likely objection at this point is that not all patients will be saved by the preventive measures just mentioned. But then the answer should be to further *improve* these preventive measures, thus making amputations redundant. We should not put all patients’ bodily integrity in jeopardy just to accommodate the very few patients who (allegedly) insist on amputations.

## Implicit pressure

Worse still, requests for amputations, rather than being forced on patients for budgetary reasons, will emerge due to a more subtle kind of pressure. The mere option of amputation coerces patients with maybe not so serious infections etc. to consider this easy and – admittedly – effective solution. Instead of a situation in which patients will just have to have faith in ordinary medical treatment, we now have a situation of forced decision for or against amputation, a type of situation aptly described as the “prison of freedom” (Hartling 2021b). In addition, many patients with chronic and complex health problems e.g., stubborn infections, worry about being a burden to their families and the health care system due to lengthy hospital admissions. Even if expectations to opt for amputation are not explicitly spelled out, patients may still (as pointed out by Sulmasy in the context of euthanasia) “introject the external cues from the physician that urge their election of this ‘right’” (Sulmasy 2017: 54). The practice of amputation creates an atmosphere in which it may not even occur to patients to say no thanks. A prohibition on amputations would in fact give such vulnerable patients a helping hand and communicate to them that they are welcome to be a burden.

## Illusions of autonomy

It seems natural to derive a right to mutilation from the principle of respect for autonomy. However, just as it is the case with euthanasia and the alleged “right to die”, autonomy is often an illusion in circumstances where the patient’s request should be heard as a cry for help rather than a rational, well-considered decision. It is a condition for autonomy that the person is well-informed and competent, but it is hard to see how one can be competent in a condition of pain and anxiety typical of situations in which one’s mobility is at stake (see Campbell 1999 for an analogous argument). And it is equally problematic to suppose that one can be well informed about a choice, the consequences of which (to be dismembered for life), no-one can imagine in advance.

## The slippery slope

Proponents of a right to mutilation insist that such a drastic measure should of course be restricted to the very serious cases where other medical interventions are ineffective and on the condition of informed consent from the patient. As already stated, however, it is doubtful if the practice can in the long run be restricted to such acute cases. Patients may gradually opt for amputations “just in case” or due to the internalized external pressure mentioned above. There are already several well-documented cases of patients who had the wrong leg amputated and it is predictable that more and more not so careful doctors abuse the option to amputate to dispose of complicated patients, to fulfil quota or out of simple neglect. The requirement of voluntariness and informed consent may also very well be gradually diluted. There are already cases of amputations without explicit consent from the patient because the patient is in a coma or otherwise unconscious or unresponsive (for similar evidence concerning euthanasia, see Pereira 2011). Who is to say that we will not gradually go from accepting such *non*voluntary to *in*voluntary mutilations?

## Conclusion

Just as there are strong reasons to doubt the moral justifiability of euthanasia, there are strong reasons to doubt whether a legally endorsed practice of amputations in health care is morally justified. Just as there should be no “right to die” there should be no right to mutilation. In the words of Leon Kass, if medicine were to renounce mutilation in all its forms, it “may serve not only the good of its patients, but also, by example, the failing moral health of modern times” (Kass 1989: 46).

## Objections to the analogy

I take it that most people find the eight argumentations above absurd or at least far from providing good reasons for an absolute prohibition of amputations (“mutilations”). So, here is the challenge: Do analogous argumentations about the pitfalls of euthanasia fare any better? They may do so if one can establish morally relevant *disanalogies* between amputations and euthanasia.

The most obvious disanalogy seems to be that amputations are (normally) motivated by a desire to *preserve life*, whereas euthanasia is motivated by the exact opposite: to *end life*. Harm to a person is thus typically considered only *prima facie* wrong. There are different versions of this idea. For instance, consequentialists could argue that the disvalue of harm is here outweighed by the positive value of saving life whereas deontologists could argue that the duty not to harm can be outweighed by a more stringent moral duty to save life. Amputations is a choice of the lesser evil. Further, an amputated (“mutilated”) person is still a person, somebody who have had a drastic solution to a problem of that person. In euthanasia, by contrast, the “solution” to the person’s problem is to eliminate the person. So, even if amputation is an extreme procedure with very high stakes, there is a positive gain in continued life and future well-being. In that sense euthanasia belongs to a different ethical category.

I do not think that this move is as strong as it may appear. First, it postulates a false disanalogy in the motivations for amputations and euthanasia: preserving life versus ending life. But as I have indicated in Sects. 1 and 2 above, to claim that euthanasia is motivated by a desire to end life is precisely analogous to the absurd claim that amputations are motivated by a desire to mutilate. Neither does euthanasia involve a decision to eliminate the person. In standard requests for euthanasia that “decision” has already been made by the fact of terminal illness. In such cases euthanasia is a choice of *the manner of one’s dying*, not a “choice of death”. Secondly, paradigmatic euthanasia cases are situations in which saving life is either futile anyway or of negative rather than positive value. Unless one ascribes unconditional value to continued biological life, no matter under which conditions, euthanasia can be a choice of the lesser evil quite analogous to amputations, given that the alternative is unbearable and pointless suffering. Analogous to the deontological reasoning, one may point to the idea of human life as a unique combination of biology and biography; of being a highly complex living organism and a lived life or existence constituted by unique relationships, projects, commitments, and attachments (Dworkin 1993). Deontologists could argue that the duty to respect a person’s biographical life is at least sometimes more stringent than the duty to preserve mere biological life, especially if it is the case that biological life can be preserved only for days or weeks.

I conclude that even if it is quite plausible to find amputations less troubling than euthanasia, I still think that the objections against euthanasia hold the analogous implications for amputations to some degree and thus lends the analogy ad absurdum good if not compelling reasons to be skeptical about the stock arguments against euthanasia. The challenge still stands.
